# Supplementary material for: WDFY2 restrains matrix metalloproteinase secretion and cell invasion by controlling VAMP3-dependent recycling
Source: Nat Commun. 2019 Jun 28;10:2850. doi: 10.1038/s41467-019-10794-w (PMC6599030; doi:10.1038/s41467-019-10794-w)
Supplement: Supplementary file 2 — Reporting Summary [file 41467_2019_10794_MOESM2_ESM.pdf]

nature research

Corresponding author(s): Kay Oliver Schink
Harald Stenmark

Last updated by author(s): Apr 11, 2019

Reporting Summary

Nature Research wishes to improve the reproducibility of the work that we publish. This form provides structure for consistency and transparency in reporting. For further information on Nature Research policies, see Authors & Referees and the Editorial Policy Checklist.

Statistics

For all statistical analyses, confirm that the following items are present in the figure legend, table legend, main text, or Methods section.

n/a	Confirmed	
☐	☒	The exact sample size (*n*) for each experimental group/condition, given as a discrete number and unit of measurement
☐	☒	A statement on whether measurements were taken from distinct samples or whether the same sample was measured repeatedly
☐	☒	The statistical test(s) used AND whether they are one- or two-sided<br>*Only common tests should be described solely by name; describe more complex techniques in the Methods section.*
☒	☐	A description of all covariates tested
☐	☒	A description of any assumptions or corrections, such as tests of normality and adjustment for multiple comparisons
☐	☒	A full description of the statistical parameters including central tendency (e.g. means) or other basic estimates (e.g. regression coefficient) AND variation (e.g. standard deviation) or associated estimates of uncertainty (e.g. confidence intervals)
☐	☒	For null hypothesis testing, the test statistic (e.g. *F*, *t*, *r*) with confidence intervals, effect sizes, degrees of freedom and *P* value noted<br>*Give P values as exact values whenever suitable.*
☒	☐	For Bayesian analysis, information on the choice of priors and Markov chain Monte Carlo settings
☒	☐	For hierarchical and complex designs, identification of the appropriate level for tests and full reporting of outcomes
☒	☐	Estimates of effect sizes (e.g. Cohen's *d*, Pearson's *r*), indicating how they were calculated

Our web collection on statistics for biologists contains articles on many of the points above.

Software and code

Policy information about availability of computer code

Data collection

Image acquisition was performed using Softworx software (GE Healthcare), Zen Software (Zeiss), and ScanR software (Olympus).

Data analysis

Deconvolution was performed using Softworx software and PRIISM ER-Decon software.
Imaging data was analyzed using FIJI/Imagej and postprocessed using Python scripts.
dSTORM/PAINT data was analyzed using Softworx software and ImageJ.
Proteomic analysis was performed using MaxQuant software.
High throughput imaging data was analzyed using ScanR software.
Data analysis, statistics and plotting was performed using Graphpad Prism and Python Pandas / Seaborn modules. The used scripts have been deposited on GitHub (at https://github.com/koschink/Sneeggen_et_al) .

For manuscripts utilizing custom algorithms or software that are central to the research but not yet described in published literature, software must be made available to editors/reviewers. We strongly encourage code deposition in a community repository (e.g. GitHub). See the Nature Research guidelines for submitting code & software for further information.

Data

Policy information about availability of data

All manuscripts must include a data availability statement. This statement should provide the following information, where applicable:

- Accession codes, unique identifiers, or web links for publicly available datasets
- A list of figures that have associated raw data
- A description of any restrictions on data availability

The data that support the findings of this study are available from the corresponding authors (H.S, K.O.S) upon reasonable request. Raw data and python scripts to

recreate shown plots are available from GitHub (https://github.com/koschink/Sneeggen_et_al)

Field-specific reporting

Please select the one below that is the best fit for your research. If you are not sure, read the appropriate sections before making your selection.

☒ Life sciences ☐ Behavioural & social sciences ☐ Ecological, evolutionary & environmental sciences

For a reference copy of the document with all sections, see nature.com/documents/nr-reporting-summary-flat.pdf

Life sciences study design

All studies must disclose on these points even when the disclosure is negative.

Sample size	Sample size was not predetermined, instead, efforts were made for each experiment to generate the the highest n values that could reasonably achieved.
Data exclusions	Collected data was only excluded for technical reasons:- if verification of knockdown efficiency showed insufficient knockdown (less than 70 %). - Live-cell imaging data was excluded if it showed strong photobleaching or the imaged cells showed phototoxicity effects; in these cases, samples were excluded prior to analysis.
Replication	All attempts at replication were successful.
Randomization	This study used cultured cell lines which are homogenous and isogenic, therefore, no randomization was used prior to treatment.
Blinding	For Gelatin degradation assays, both image acquisition and analysis were performed blinded. The other experiments were performed non-blinded; however, efforts were made to analyze quantitative experiments without user intervention, e.g. by using automated image scoring by ScanR software.

Reporting for specific materials, systems and methods

We require information from authors about some types of materials, experimental systems and methods used in many studies. Here, indicate whether each material, system or method listed is relevant to your study. If you are not sure if a list item applies to your research, read the appropriate section before selecting a response.

Materials & experimental systems

n/a	Involved in the study	
☐	☒	Antibodies
☐	☒	Eukaryotic cell lines
☐	☐	Palaeontology
☐	☐	Animals and other organisms
☐	☐	Human research participants
☐	☐	Clinical data

Methods

n/a	Involved in the study	
☐	☐	ChIP-seq
☐	☐	Flow cytometry
☐	☐	MRI-based neuroimaging

Antibodies

Antibodies used

Primary antibodies:
Anti-EEA1 provided by Ban-Hock Toh, Monash university, Human (1/160,000)
Anti-APPL1 D83H4 XP®, cell signaling (3858S), Rabbit (1/100)
Anti-Rab7(D95F2) XP®, Cell signaling (9367), Rabbit (1/200)
Anti-RAB11, Polyclonal, Invitrogen™, Zymed laboratories (71-5300), Rabbit (1/100).
Anti-Rab5 (4F11) was a gift from C. Bucci , University of Lecce, Italy, Mouse (1/2500), Wenzel et.al 2018.
Anti-Rab4, Polyclonal, Invitrogen™, Fisher Scientific (PA3-912), Rabbit (1/200).
anti-GFP Monoclonal, Roche (11814460001), mouse (1:400).
RFP booster ATTO-594, Chromotek (rba594)(1/500).
Anti-HRS described previously, Rabbit (1/100), Raiborg et.al 2001
Anti-LAMP1 antibody produced in rabbit, Sigma-Aldrich (L1418), Rabbit (1/200).
Anti-VAMP 1/2/3, Synaptic Systems (104-203), Rabbit (1/200)
Anti-MMP-14 Antibody, catalytic domain, clone LEM-2/15.8, Merck Life science (MAB3328), Mouse (1/800).
Anti-TGN46 antibody, AbD Serotec (AHP500G), Sheep (1/100).
Monoclonal Anti-γ-Tubulin antibody produced in mouse, clone GTU-88, Sigma Aldrich (T6557).
Monoclonal Anti-α-Tubulin antibody produced in mouse (T5168)
Anti-VPS35 antibody, Abcam (ab10099), Goat (1/100).
Anti-VPS26 antibody, Abcam (ab23892), Rabbit (1/100).
Anti-beta Tubulin antibody – Loading Control, Abcam (ab6046).
Anti-mCherry, Acris Antibodies (AB0040-200), Goat (1/100).

Anti-GST HRP Conjugate, GE Healthcare (RPN1236). (1/5000).

Secondary:
Alexa Fluor 647 Donkey anti-Mouse, Jackson Immuno Research (715-605-150), (1/500)
Alexa Fluor 647 Donkey anti- Human, Jackson Immuno Research (709-605-149), (1/500).
Alexa Fluor 488 Donkey anti-Mouse, Jackson Immuno Resarch (715 545 151), (1/500).
Alexa Fluor 555 Donkey anit-Mouse, Life Technologies (A31570), (1/500).
Alexa Fluor 555 Donkey anti-Rabbit, Life Technologies (A31572), (1/500).
Alexa Fluor 555 Donkey anti-Goat, Life Technologies (A21432), (1/500).
Alexa Fluor 568 Donkey anti-Sheep, Life Technologies (A-21099), (1/500).

Donkey Anti-Rabbit IRDye 800CW, Li-Cor (926-32213), (1/10000).
Donkey Anti-Mouse IRDye 800CW IgG (H+L), LiCor (926-32212), (1/10000)
Donkey Anti-Mouse IRDye 680, LiCor (926-32222), (1/10000)
Donkey Anti-Rabbit IRDye 680RD, LiCor (926-68073), (1/10000)

Validation

Primary antibodies:
Anti-EEA1 provided by Ban-Hock Toh, Monash university, Human (1/160,000), described in Raiborg et.al 2001
Anti-APPL1 D83H4 XP®, cell signaling (3858S), Rabbit (1/100), Manufacturers data sheet.
Anti-Rab7(D95F2) XP®, Cell signaling (9367), Rabbit (1/200), Manufacturers data sheet.
Anti-RAB11, Polyclonal, Invitrogen™, Zymed laboratories (71-5300), Rabbit (1/100), Manufacturers data sheet.
Anti-Rab5 (4F11) was a gift from C. Bucci , University of Lecce, Italy, Mouse (1/2500), Wenzel et.al 2018.
Anti-Rab4, Polyclonal, Invitrogen™, Fisher Scientific (PA3-912), Rabbit (1/200), Manufacturers data sheet.
anti-GFP Monoclonal, Roche (11814460001), mouse (1:400), Manufacturers data sheet
RFP booster ATTO-594, Chromotek (rba594)(1/500). Manufacturers data sheet.
Anti-HRS described previously, Rabbit (1/100), Raiborg et.al 2001
Anti-LAMP1 antibody produced in rabbit, Sigma-Aldrich (L1418), Rabbit (1/200). Manufacturers data sheet.
Anti-VAMP 1/2/3, Synaptic Systems (104-203), Rabbit (1/200), Validated by siRNA knockdown.
Anti-MMP-14 Antibody, catalytic domain, clone LEM-2/15.8, Merck Life science (MAB3328), Mouse (1/800), Validated by siRNA knockdown.
Anti-TGN46 antibody, AbD Serotec (AHP500G), Sheep (1/100). Manufacturers data sheet.
Monoclonal Anti-γ-Tubulin antibody produced in mouse, clone GTU-88, Sigma Aldrich (T6557). Manufacturers data sheet.
Anti-VPS35 antibody, Abcam (ab10099), Goat (1/100). Manufacturers data sheet.
Anti-VPS26 antibody, Abcam (ab23892), Rabbit (1/100). Manufacturers data sheet.
Anti-beta Tubulin antibody – Loading Control, Abcam (ab6046). Manufacturers data sheet.
Anti-mCherry, Acris Antibodies (AB0040-200), Goat (1/100). Manufacturers data sheet.
Anti-GST HRP Conjugate, GE Healthcare (RPN1236). (1/5000). Manufacturers data sheet.

Eukaryotic cell lines

Policy information about cell lines

Cell line source(s)

hTERT-RPE1 cells, PC3 cells and MDA-MB321 cells were purchased from ATCC.

Authentication

Cells were authenticated by the Genotyping core facility at Oslo University hospital using Powerplex16 assays

Mycoplasma contamination

Original cell stocks were verified to be mycoplasma-free and were regularly tested, after manipulation (e.g. generation of clones or cell lines), cells were again verified to be mycoplasma-free.

Commonly misidentified lines
(See ICLAC register)

Name any commonly misidentified cell lines used in the study and provide a rationale for their use.

Palaeontology

Specimen provenance

Provide provenance information for specimens and describe permits that were obtained for the work (including the name of the issuing authority, the date of issue, and any identifying information).

Specimen deposition

Indicate where the specimens have been deposited to permit free access by other researchers.

Dating methods

If new dates are provided, describe how they were obtained (e.g. collection, storage, sample pretreatment and measurement), where they were obtained (i.e. lab name), the calibration program and the protocol for quality assurance OR state that no new dates are provided.

☐ Tick this box to confirm that the raw and calibrated dates are available in the paper or in Supplementary Information.

Animals and other organisms

Policy information about studies involving animals; ARRIVE guidelines recommended for reporting animal research

Laboratory animals

For laboratory animals, report species, strain, sex and age OR state that the study did not involve laboratory animals.

Wild animals	*Provide details on animals observed in or captured in the field; report species, sex and age where possible. Describe how animals were caught and transported and what happened to captive animals after the study (if killed, explain why and describe method; if released, say where and when) OR state that the study did not involve wild animals.*
Field-collected samples	*For laboratory work with field-collected samples, describe all relevant parameters such as housing, maintenance, temperature, photoperiod and end-of-experiment protocol OR state that the study did not involve samples collected from the field.*
Ethics oversight	*Identify the organization(s) that approved or provided guidance on the study protocol, OR state that no ethical approval or guidance was required and explain why not.*

Note that full information on the approval of the study protocol must also be provided in the manuscript.

Human research participants

Policy information about studies involving human research participants

Population characteristics	*Describe the covariate-relevant population characteristics of the human research participants (e.g. age, gender, genotypic information, past and current diagnosis and treatment categories). If you filled out the behavioural & social sciences study design questions and have nothing to add here, write "See above."*
Recruitment	*Describe how participants were recruited. Outline any potential self-selection bias or other biases that may be present and how these are likely to impact results.*
Ethics oversight	*Identify the organization(s) that approved the study protocol.*

Note that full information on the approval of the study protocol must also be provided in the manuscript.

Clinical data

Policy information about clinical studies

All manuscripts should comply with the ICMJE guidelines for publication of clinical research and a completed CONSORT checklist must be included with all submissions.

Clinical trial registration	*Provide the trial registration number from ClinicalTrials.gov or an equivalent agency.*
Study protocol	*Note where the full trial protocol can be accessed OR if not available, explain why.*
Data collection	*Describe the settings and locales of data collection, noting the time periods of recruitment and data collection.*
Outcomes	*Describe how you pre-defined primary and secondary outcome measures and how you assessed these measures.*

ChIP-seq

Data deposition

☐ Confirm that both raw and final processed data have been deposited in a public database such as GEO.

☐ Confirm that you have deposited or provided access to graph files (e.g. BED files) for the called peaks.

Data access links<br>*May remain private before publication.*	*For "Initial submission" or "Revised version" documents, provide reviewer access links. For your "Final submission" document, provide a link to the deposited data.*
Files in database submission	*Provide a list of all files available in the database submission.*
Genome browser session<br>(e.g. UCSC)	*Provide a link to an anonymized genome browser session for "Initial submission" and "Revised version" documents only, to enable peer review. Write "no longer applicable" for "Final submission" documents.*

Methodology

Replicates	*Describe the experimental replicates, specifying number, type and replicate agreement.*
Sequencing depth	*Describe the sequencing depth for each experiment, providing the total number of reads, uniquely mapped reads, length of reads and whether they were paired- or single-end.*
Antibodies	*Describe the antibodies used for the ChIP-seq experiments; as applicable, provide supplier name, catalog number, clone name, and lot number.*
Peak calling parameters	*Specify the command line program and parameters used for read mapping and peak calling, including the ChIP, control and index files used.*
Data quality	*Describe the methods used to ensure data quality in full detail, including how many peaks are at FDR 5% and above 5-fold enrichment.*

Software | *Describe the software used to collect and analyze the ChIP-seq data. For custom code that has been deposited into a community repository, provide accession details.*

Flow Cytometry

Plots

Confirm that:

- [ ] The axis labels state the marker and fluorochrome used (e.g. CD4-FITC).
- [ ] The axis scales are clearly visible. Include numbers along axes only for bottom left plot of group (a 'group' is an analysis of identical markers).
- [ ] All plots are contour plots with outliers or pseudocolor plots.
- [ ] A numerical value for number of cells or percentage (with statistics) is provided.

Methodology

Sample preparation | *Describe the sample preparation, detailing the biological source of the cells and any tissue processing steps used.*

Instrument | *Identify the instrument used for data collection, specifying make and model number.*

Software | *Describe the software used to collect and analyze the flow cytometry data. For custom code that has been deposited into a community repository, provide accession details.*

Cell population abundance | *Describe the abundance of the relevant cell populations within post-sort fractions, providing details on the purity of the samples and how it was determined.*

Gating strategy | *Describe the gating strategy used for all relevant experiments, specifying the preliminary FSC/SSC gates of the starting cell population, indicating where boundaries between "positive" and "negative" staining cell populations are defined.*

- [ ] Tick this box to confirm that a figure exemplifying the gating strategy is provided in the Supplementary Information.

Magnetic resonance imaging

Experimental design

Design type | *Indicate task or resting state; event-related or block design.*

Design specifications | *Specify the number of blocks, trials or experimental units per session and/or subject, and specify the length of each trial or block (if trials are blocked) and interval between trials.*

Behavioral performance measures | *State number and/or type of variables recorded (e.g. correct button press, response time) and what statistics were used to establish that the subjects were performing the task as expected (e.g. mean, range, and/or standard deviation across subjects).*

Acquisition

Imaging type(s) | *Specify: functional, structural, diffusion, perfusion.*

Field strength | *Specify in Tesla*

Sequence & imaging parameters | *Specify the pulse sequence type (gradient echo, spin echo, etc.), imaging type (EPI, spiral, etc.), field of view, matrix size, slice thickness, orientation and TE/TR/flip angle.*

Area of acquisition | *State whether a whole brain scan was used OR define the area of acquisition, describing how the region was determined.*

Diffusion MRI ☐ Used ☐ Not used

Preprocessing

Preprocessing software | *Provide detail on software version and revision number and on specific parameters (model/functions, brain extraction, segmentation, smoothing kernel size, etc.).*

Normalization | *If data were normalized/standardized, describe the approach(es): specify linear or non-linear and define image types used for transformation OR indicate that data were not normalized and explain rationale for lack of normalization.*

Normalization template | *Describe the template used for normalization/transformation, specifying subject space or group standardized space (e.g. original Talairach, MNI305, ICBM152) OR indicate that the data were not normalized.*

Noise and artifact removal	*Describe your procedure(s) for artifact and structured noise removal, specifying motion parameters, tissue signals and physiological signals (heart rate, respiration).*
Volume censoring	*Define your software and/or method and criteria for volume censoring, and state the extent of such censoring.*

Statistical modeling & inference

Model type and settings	*Specify type (mass univariate, multivariate, RSA, predictive, etc.) and describe essential details of the model at the first and second levels (e.g. fixed, random or mixed effects; drift or auto-correlation).*
Effect(s) tested	*Define precise effect in terms of the task or stimulus conditions instead of psychological concepts and indicate whether ANOVA or factorial designs were used.*

Specify type of analysis: ☐ Whole brain ☐ ROI-based ☐ Both

Statistic type for inference<br>(See Eklund et al. 2016)	*Specify voxel-wise or cluster-wise and report all relevant parameters for cluster-wise methods.*
Correction	*Describe the type of correction and how it is obtained for multiple comparisons (e.g. FWE, FDR, permutation or Monte Carlo).*

Models & analysis

n/a	Involved in the study
☐	☐ Functional and/or effective connectivity
☐	☐ Graph analysis
☐	☐ Multivariate modeling or predictive analysis

Functional and/or effective connectivity	*Report the measures of dependence used and the model details (e.g. Pearson correlation, partial correlation, mutual information).*
Graph analysis	*Report the dependent variable and connectivity measure, specifying weighted graph or binarized graph, subject- or group-level, and the global and/or node summaries used (e.g. clustering coefficient, efficiency, etc.).*
Multivariate modeling and predictive analysis	*Specify independent variables, features extraction and dimension reduction, model, training and evaluation metrics.*
