# Supplementary material for: WDFY2 restrains matrix metalloproteinase secretion and cell invasion by controlling VAMP3-dependent recycling
Source: Nat Commun. 2019 Jun 28;10:2850. doi: 10.1038/s41467-019-10794-w (PMC6599030; doi:10.1038/s41467-019-10794-w)
Supplement: Supplementary file 3 — Description of Additional Supplementary Files [file 41467_2019_10794_MOESM3_ESM.pdf]

Description of Additional Supplementary Files

File Name: Supplementary Movie 1
Description: Deconvolved widefield time-lapse showing endosome labelled with GFP-WDFY2 and endosomal tubules positive for WDFY2. Images were acquired every 2s. Scale bar: 1 µm.

File Name: Supplementary Movie 2
Description: Deconvolved widefield time-lapse imaging showing endogenously NLAP-tagged WDFY2 localizing to tubules on EEA1-labelled endosomes. Images were acquired very second. Scale bar: 5 µm

File Name: Supplementary Movie 3
Description: Deconvolved widefield time-lapse showing endosome labelled with GFP-WDFY2 and mCherry-Coronin1B. Images were acquired every 2s. Scale bar: 1 µm.

File Name: Supplementary Movie 4
Description: Deconvolved widefield time-lapse showing endosomes and endosomal tubules labelled with GFP-WDFY2 prior to addition of Latrunculin and after addition. Images were acquired every 3s. Scale bar:10 µm.

File Name: Supplementary Movie 5
Description: Deconvolved widefield time-lapse showing endosomes and endosomal tubules labelled with GFP-WDFY2 and PA-mCherrry-VAMP3 following photoactivation. Images were acquired every second.

File Name: Supplementary Movie 6
Description: TIRF time-lapse movie showing secretion events of pHluorin-Vamp3 in WT and WDFY2(-/-) cells. Images were acquired every second. Scale bar: 10 µm, inset: 1 µm.

File Name: Supplementary Movie 7
Description: Deconvolved widefield time-lapse movie showing endosomal tubules – visualized by mCherry-2xFYVEWDFY2 – in wild-type hTERT-RPE1 cells. Images were acquired every second. Scale bar: 1µm.

File Name: Supplementary Movie 8
Description: Deconvolved widefield time-lapse movie showing endosomal tubules – visualized by mCherry-2xFYVEWDFY2 – in WDFY2(-/-) cells. Images were acquired every second. Scale bar: 1µm.

File Name: Supplementary Movie 9
Description: Deconvolved widefield time-lapse movie showing localization of GFP-MT1-MMP and mCherry-WDFY2 on endosomes and endosomal tubules. Images were acquired every second. Scale bar: 1µm.

File Name: Supplementary Movie 10
Description: Deconvolved widefield time-lapse movie showing localization of GFP-MT1-MMP and mCherry-Rab4 on endosomes and endosomal tubules. Images were acquired every second. Scale bar: 1µm.

File Name: Supplementary Movie 11
Description: Deconvolved widefield time-lapse movie showing localization of GFP-WDFY2 and mCherry-Rab4 on endosomes and endosomal tubules. Images were acquired every second. Scale bar: 1µm.

File Name: Supplementary Movie 12
Description: TIRF time-lapse movie showing exocytosis events of pHluorin-MT1-MMP in WT and WDFY2(-/-) cells. Images were acquired every second. Scale bar: 10 µm, inset: 1 µm.

File Name: Supplementary Data 1
Description: List of identified WDFY2 interactors
