# Supplementary material for: WDFY2 restrains matrix metalloproteinase secretion and cell invasion by controlling VAMP3-dependent recycling
Source: Nat Commun. 2019 Jun 28;10:2850. doi: 10.1038/s41467-019-10794-w (PMC6599030; doi:10.1038/s41467-019-10794-w)
Supplement: Supplementary file 17 — Supplementary information [file 41467_2019_10794_MOESM17_ESM.pdf]

Supplementary Information

WDFY2 restrains matrix metalloproteinase secretion and cell invasion by controlling VAMP3-dependent recycling

Sneeggen et al.

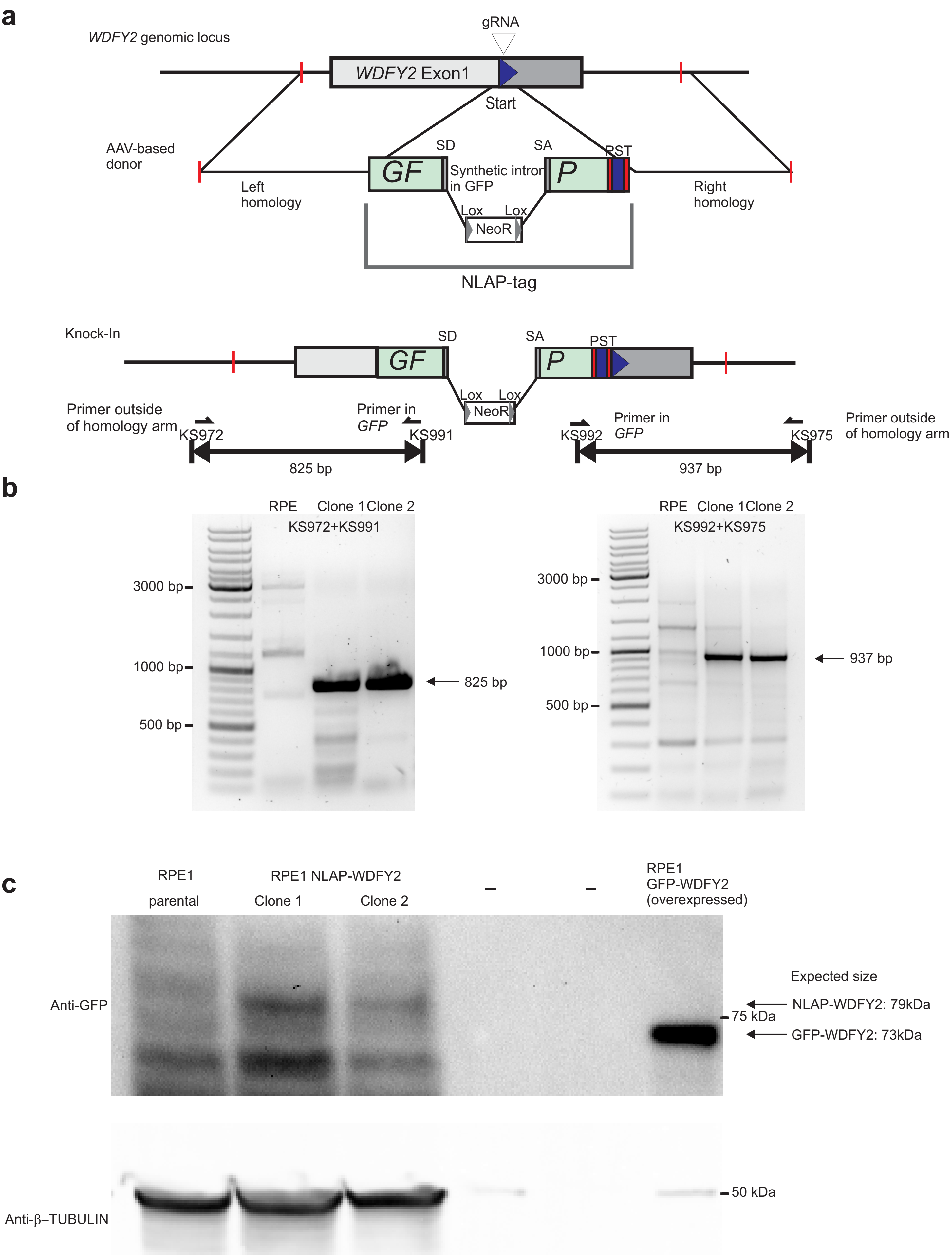
a
WDFY2 genomic locus
gRNA
WDFY2 Exon1
Start
AAV-based donor
Left homology
GF
SD
Synthetic intron in GFP
SA
P
PST
Right homology
Lox
Lox
NeoR
NLAP-tag
Knock-In
GF
SD
SA
P
PST
Lox
Lox
NeoR
Primer outside of homology arm
KS972
Primer in GFP
KS991
825 bp
KS992
Primer in GFP
KS975
Primer outside of homology arm
937 bp
b
RPE
Clone 1
Clone 2
KS972+KS991
3000 bp
1000 bp
500 bp
825 bp
RPE
Clone 1
Clone 2
KS992+KS975
3000 bp
1000 bp
500 bp
937 bp
c
RPE1 parental
RPE1 NLAP-WDFY2
Clone 1
Clone 2
–
–
RPE1 GFP-WDFY2 (overexpressed)
Anti-GFP
Expected size
NLAP-WDFY2: 79kDa
75 kDa
GFP-WDFY2: 73kDa
Anti-β–TUBULIN
50 kDa

Supplementary Figure1: Generation and characterization of endogenously tagged WDFY2

a) Tagging strategy to generate endogenously tagged WDFY2. Shown are the endogenous locus, the homology donor construct, the expected outcome after homologous recombination, the binding site of gRNAs and verification primers. SD=Splice donor, SA=splice acceptor, PST = Precsission-site,S-Tag,TEV-site.
b) PCR verification of the correct integration of the homology donor. Wild-type hTERT-RPE1 cells and two clones are shown. Expected sizes: 825bp and 937 bp.
c) Western blot verification of endogenous tagging of WDFY2. Shown are parental cells and two clones. Tagged WDFY2 as detected by blotting against the GFP part of the NLAP tag (expected size: 79 kDa). Overexpressed GFP-WDFY2 serves as size reference (expected size: 73 kDa), the size difference is based on different size of the used tags (NLAP vs GFP).

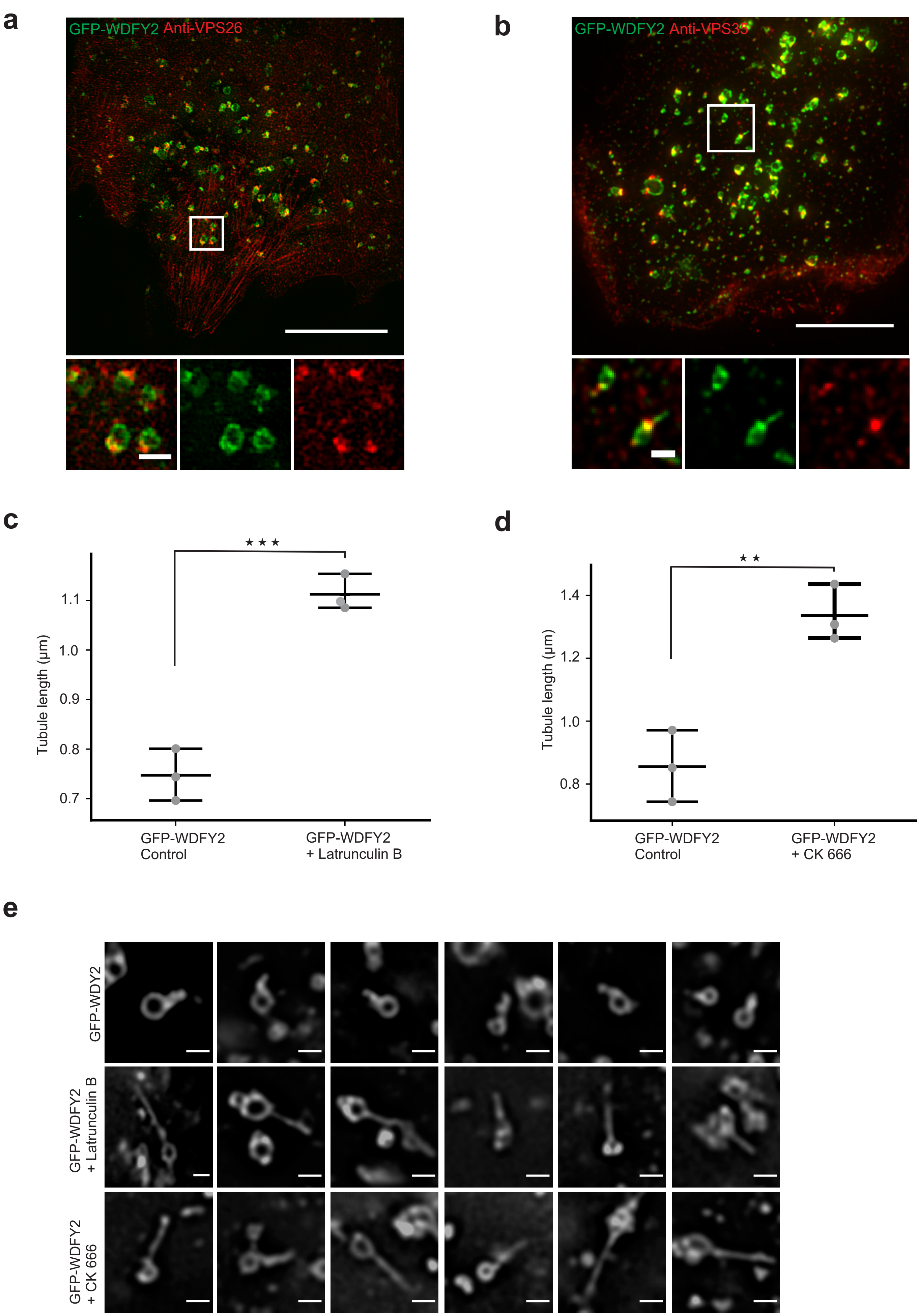
a
GFP-WDFY2 Anti-VPS26
b
GFP-WDFY2 Anti-VPS35
c
★★★
1.1
1.0
0.9
0.8
0.7
Tubule length (µm)
GFP-WDFY2
Control
GFP-WDFY2
+ Latrunculin B
d
★★
1.4
1.2
1.0
0.8
Tubule length (µm)
GFP-WDFY2
Control
GFP-WDFY2
+ CK 666
e
GFP-WDY2
GFP-WDFY2
+ Latrunculin B
GFP-WDFY2
+ CK 666

Supplementary Figure 2: Characterization of WDFY2-labelled endosomal tubules

a) SIM image of GFP-WDFY2 and VPS26, visualized with antibody, show localization to endosomes and the base of WDFY2 positive tubules. Representative image of 8 cells. Scale bar: 10 µm, inset: 1 µm.
b) Deconvolved widefield image showing GFP-WDFY2 and anti-VPS35 localization to endosomes and the base of WDFY2 positive tubules. Representative image of 8 cells. Scale bar: 10 µm, inset: 1 µm.
c) Quantifications of tubule length in hTERT-RPE1 cells stably expressing GFP-WDFY2 before and after treatment with Latrunculin B. Cells were imaged every 5 s for 20 min (n = 3 experiments, in total 255 tubules (before Latrunculin B treatment) and 200 tubules (after Latrunculin B treatment). Shown are individual experiments and the mean ± 95% CI. p =0.0020. *p < 0.05, **p < 0.01, ***p < 0.001, n.s. not statistically significant.
d) Quantifications of tubule length in hTERT-RPE1 cells stably expressing GFP-WDFY2 before and after treatment with CK666. Cells were imaged every 5 s for 20 min n = 3 experiments, in total 581 tubules (before CK666 treatment) and 578 tubules (after CK666 treatment). Shown are individual experiments and the mean ± 95% CI. p = 0.0045. *p < 0.05, **p < 0.01, ***p < 0.001, n.s. not statistically significant.
e) Deconvolved widefield image showing example images of hTERT-RPE1 cells stably expressing GFP-WDFY2 before and after treatment with Latrunculin B or CK666. Scale bar: 1 µm

Source data are provided as a Source Data file.

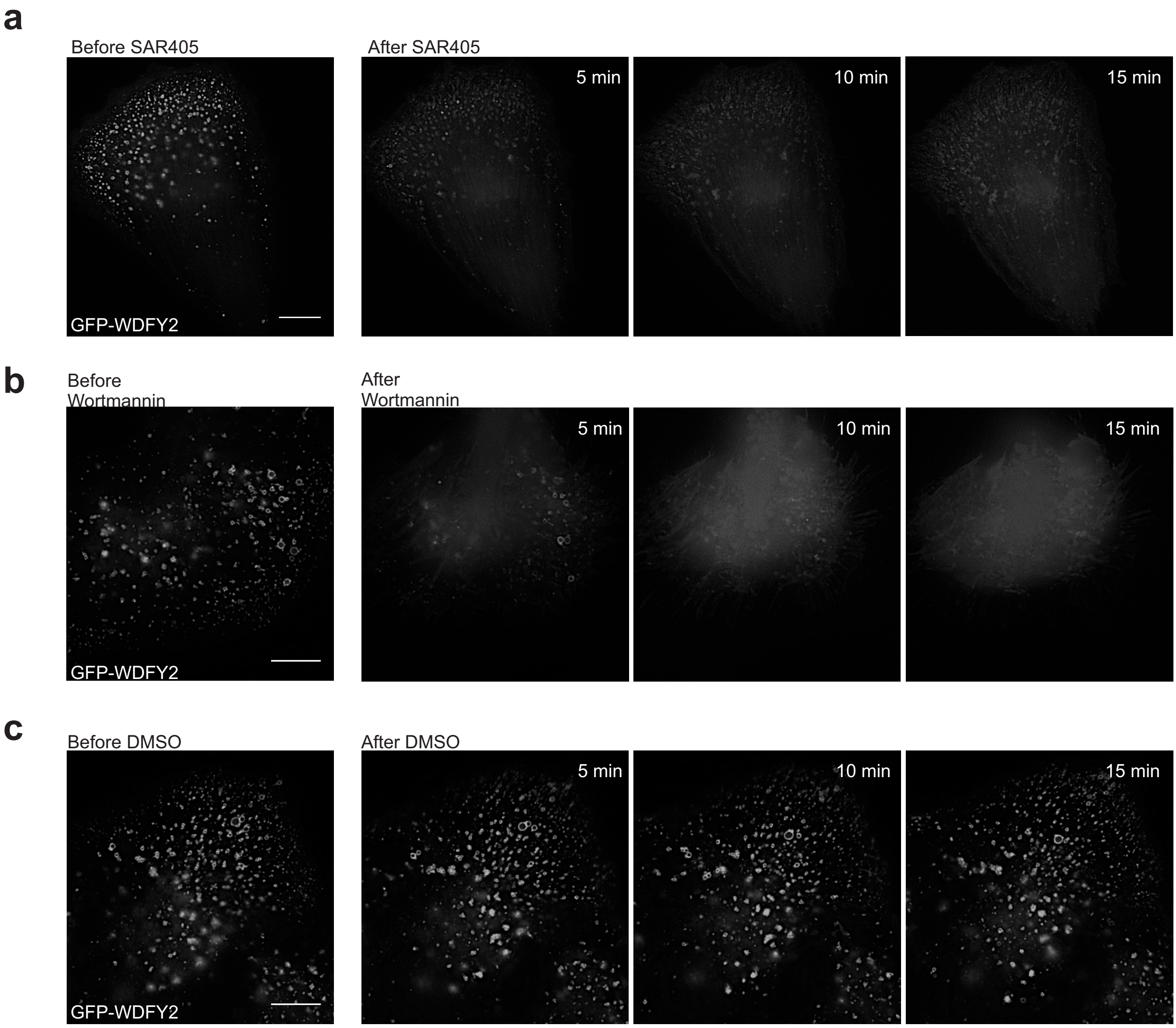
a
Before SAR405
After SAR405
5 min
10 min
15 min
GFP-WDFY2
b
Before Wortmannin
After Wortmannin
5 min
10 min
15 min
GFP-WDFY2
c
Before DMSO
After DMSO
5 min
10 min
15 min
GFP-WDFY2

Supplementary Figure 3: PtdIns3P is required for WDFY2 localization

a) Deconvolved widefield images showing hTERT-RPE1 cells stably expressing GFP-WDFY2 before and after treatment with SAR405 with a final concentration of 6 µM. After addition of SAR405 GFP-WDFY2 signal to endosomes rapidly decreases. Scale bar: 10 µm. Representative image of 5 cells.

b) Deconvolved widefield images showing hTERT-RPE1 cells stably expressing GFP-WDFY2 before and after treatment with Wortmannin with a final concentration of 6 µM. After addition of Wortmannin GFP-WDFY2 signal to endosomes rapidly decreases Scale bar: 10 µm. Representative image of 5 cells.

c) Deconvolved widefield images showing hTERT-RPE1 cells stably expressing GFP-WDFY2 before and after treatment with DMSO. Scale bar: 10 µm. Representative image of 5 cells.

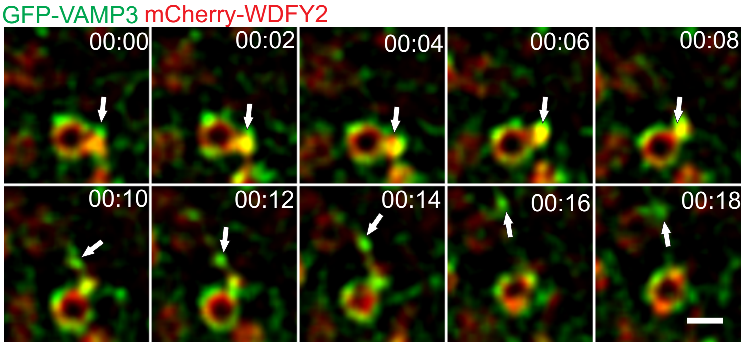
GFP-VAMP3 mCherry-WDFY2
00:00
00:02
00:04
00:06
00:08
00:10
00:12
00:14
00:16
00:18

Supplementary Figure 4: WDFY2 and VAMP3 colocalize on endosomes and endosomal tubules

Representative image sequence showing GFP-VAMP3 and mCherry-WDFY2 localization to endosomes and endosomal tubules. Shown are frames from a time-lapse movie with images acquired every 2 seconds. Representative image of 10 cells. Scale bar: 0.5 µm.

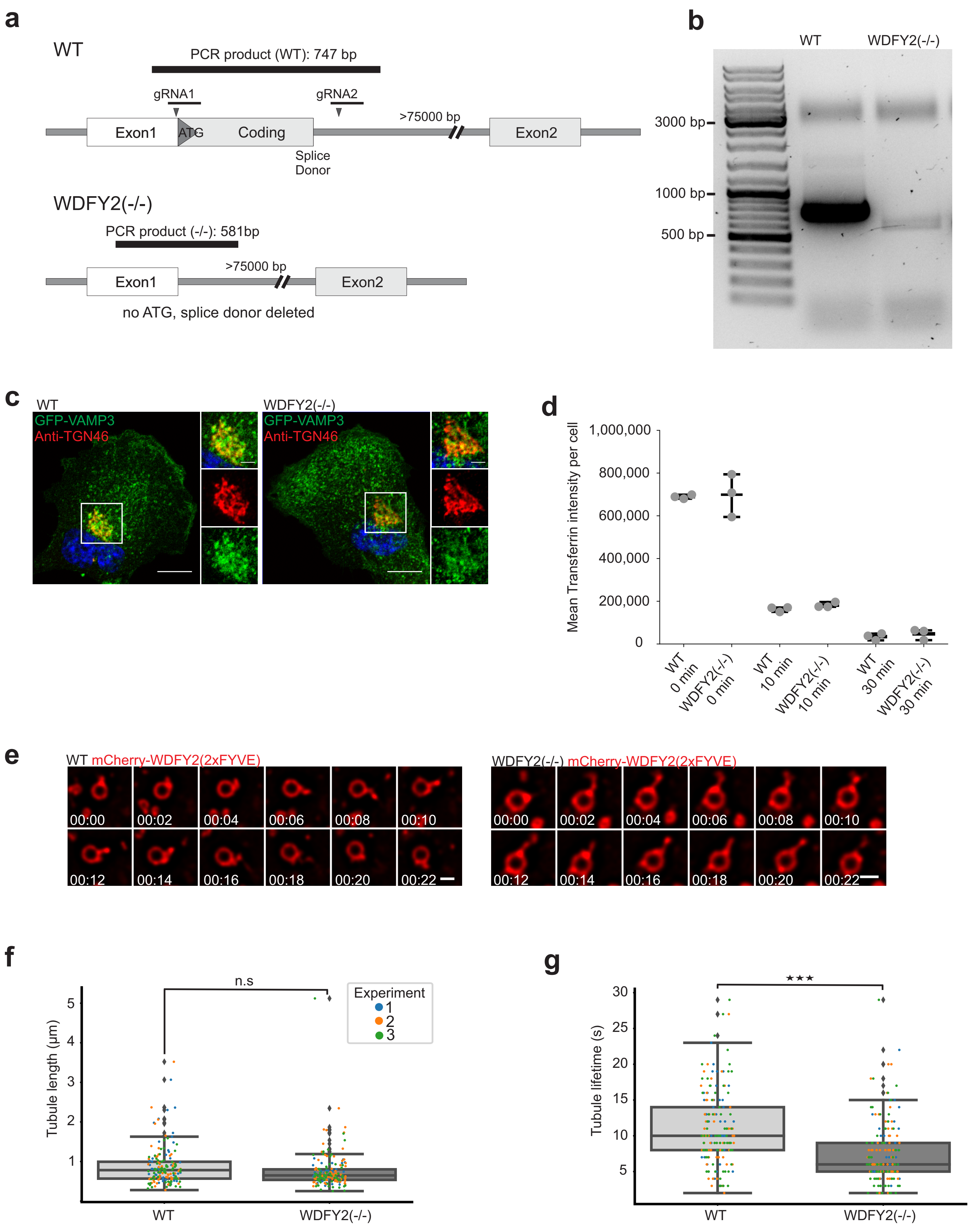
a
WT
PCR product (WT): 747 bp
gRNA1
gRNA2
>75000 bp
Exon1
ATG
Coding
Exon2
Splice Donor
WDFY2(-/-)
PCR product (-/-): 581bp
>75000 bp
Exon1
Exon2
no ATG, splice donor deleted
b
WT
WDFY2(-/-)
3000 bp
1000 bp
500 bp
c
WT
GFP-VAMP3
Anti-TGN46
WDFY2(-/-)
GFP-VAMP3
Anti-TGN46
d
Mean Transferrin intensity per cell
1,000,000
800,000
600,000
400,000
200,000
0
WT 0 min
WDFY2(-/-) 0 min
WT 10 min
WDFY2(-/-) 10 min
WT 30 min
WDFY2(-/-) 30 min
e
WT mCherry-WDFY2(2xFYVE)
WDFY2(-/-) mCherry-WDFY2(2xFYVE)
00:00
00:02
00:04
00:06
00:08
00:10
00:12
00:14
00:16
00:18
00:20
00:22
f
Tubule length (µm)
n.s
Experiment
1
2
3
WT
WDFY2(-/-)
g
Tubule lifetime (s)
***
WT
WDFY2(-/-)

Supplementary Figure 5: Generation and characterization of WDFY2 knockout cells

a) Schematic of the CRISPR/Cas9 -generated WDFY2 deletion. Indicated are location of the guide RNAs and the primers used to characterize the resulting clones. The knockout was designed that the coding region including the starting ATG and the splice donor of exon 1 was completely excised, preventing the generation of a functional mRNA.
b) PCR characterization of the CRISPR/Cas9 -generated WDFY2 deletion. Expected band sizes: Wild type: 747 bp, Knockout: 581 bp.
c) Confocal images showing localization of GFP-VAMP3 to the Golgi apparatus stained with TGN46 in WT cells and in WDFY2(-/-) cells. Representative image of 3 experiments, 10 cells per experiment and condition. Scale bar: 10µm, inset 1µm.
d) Cell-associated transferrin after recycling in WT and WDFY2(-/-) cells. The graph represents the mean intensity ± 95% CI of Alexa 488-TF per cell from 3 independent experiments. Cells analyzed in total: 3131 (WT 0 min); 2476 (WDFY2(-/-) 0 min); 3167 (WT 10 min); 2531 (WDFY2(-/-) 10 min); 2136 WT (30 min); 2292 (WDFY2(-/-) 30 min).
e) Sequential images showing WDFY2 localization to tubular structures in both WT and WDFY2(-/-) cells. Shown are frames from a time-lapse sequence with images acquired every 1 s. Scale bar: 1 µm.
f) Quantifications of tubule length (µm) in WT and WDFY2(-/-) cells shows now significant change between the wildtype and the knockout. Shown are individual data points, color-coded per experiment n=170 tubules per condition. Student's unpaired t-test, p=0.119. Shown are the median, quartiles (boxes), and 1.5 times the interquartile range (whiskers). *p < 0.05, **p < 0.01, ***p < 0.001, n.s. not statistically significant.
g) Quantifications of tubule lifetime (s) in WT and WDFY2(-/-) cells shows a significant change between the wildtype and the knockout. Shown are individual data points, color-coded per experiment n=170 tubules per condition. Student's unpaired t-test, p=0,0389. Shown are the median, quartiles (boxes), and 1.5 times the interquartile range (whiskers). *p < 0.05, **p < 0.01, ***p < 0.001, n.s. not statistically significant .

Source data are provided as a Source Data file.

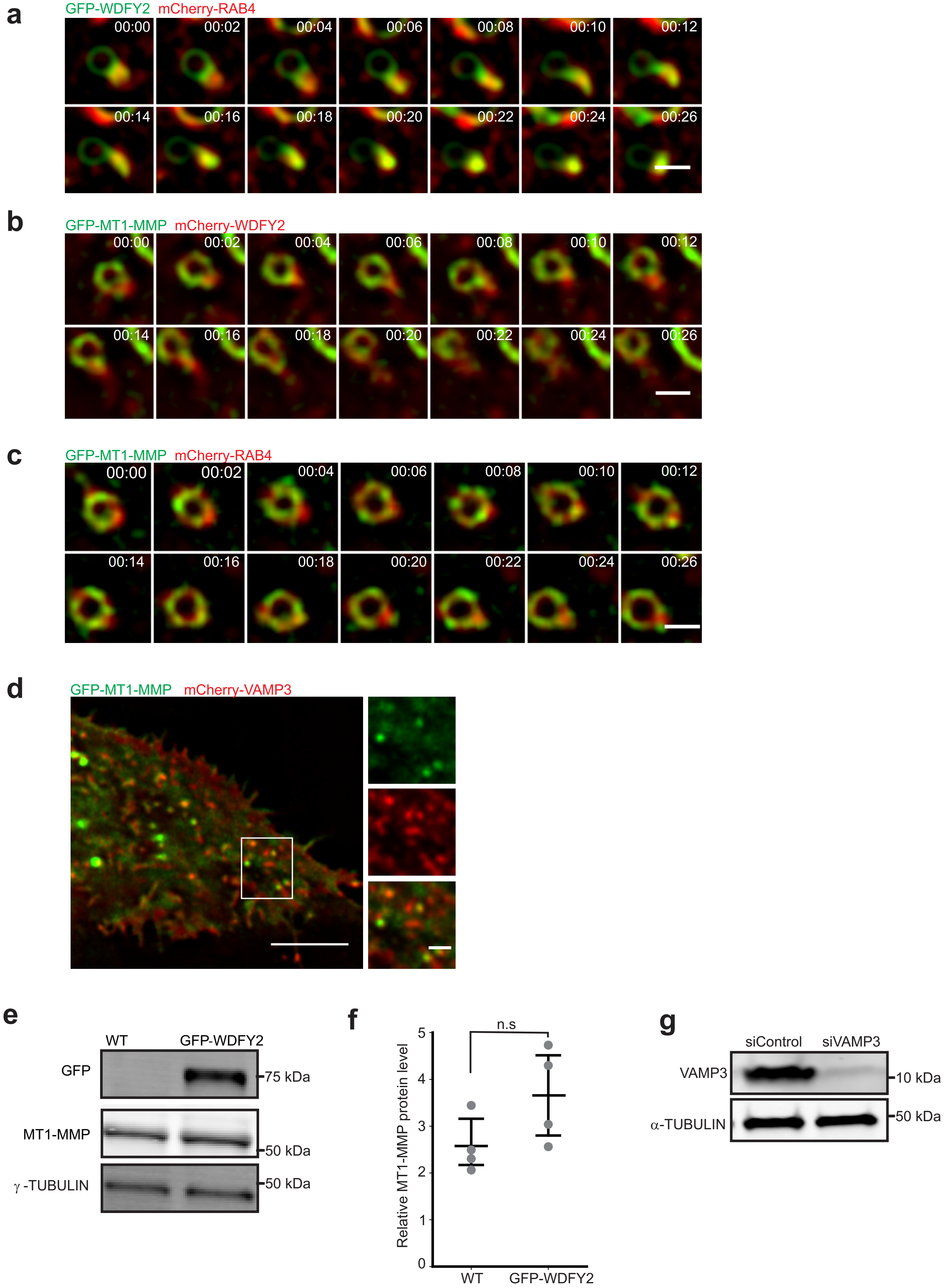
a
GFP-WDFY2 mCherry-RAB4
00:00
00:02
00:04
00:06
00:08
00:10
00:12
00:14
00:16
00:18
00:20
00:22
00:24
00:26
b
GFP-MT1-MMP mCherry-WDFY2
00:00
00:02
00:04
00:06
00:08
00:10
00:12
00:14
00:16
00:18
00:20
00:22
00:24
00:26
c
GFP-MT1-MMP mCherry-RAB4
00:00
00:02
00:04
00:06
00:08
00:10
00:12
00:14
00:16
00:18
00:20
00:22
00:24
00:26
d
GFP-MT1-MMP mCherry-VAMP3
e
WT
GFP-WDFY2
GFP
75 kDa
MT1-MMP
50 kDa
γ-TUBULIN
50 kDa
f
n.s
Relative MT1-MMP protein level
0
1
2
3
4
5
WT
GFP-WDFY2
g
siControl
siVAMP3
VAMP3
10 kDa
α-TUBULIN
50 kDa

Supplementary Figure 6: WDFY2 colocalizes with MT1-MMP, VAMP3 and Rab4 on endosomal tubules

a) Sequential images showing RAB4 localizing to WDFY2 positive tubules. Shown are frames from a time-lapse sequence with images acquired every 2 s. Scale bar: 1 µm.
b) Sequential images showing MT1-MMP localizing to WDFY2 positive tubules. Shown are frames from a time-lapse sequence with images acquired every 2 s. Scale bar: 1 µm.
c) Sequential images showing MT1-MMP localizing to RAB4 positive endosomes. Shown are frames from a time-lapse sequence with images acquired every 2 s. Scale bar: 1 µm.
d) Deconvolved widefield image showing GFP-MT1-MMP and mCherry-VAMP3 colocalization at vesicles. Scale bar: 5 µm, 1 µm (inset).
e) A representative Western blot showing MT1-MMP protein levels from WT and RPE1 cells stably expressing GFP-WDFY2 n = 4 experiments.
f) Quantification of MT1-MMP protein levels from Western blot shown in figure g show no significant change in the MT1-MMP protein level when WDFY2 is overexpressed compared to WT cells n = 4 experiments. Shown are individual data points and the mean ± 95% CI. p=0.118. n.s. not statistically significant ..
g) A representative Western blot showing VAMP3 protein levels from WDFY2(-/-) cells treated with siControl and siVAMP3 n = 4 experiments

Source data are provided as a Source Data file.

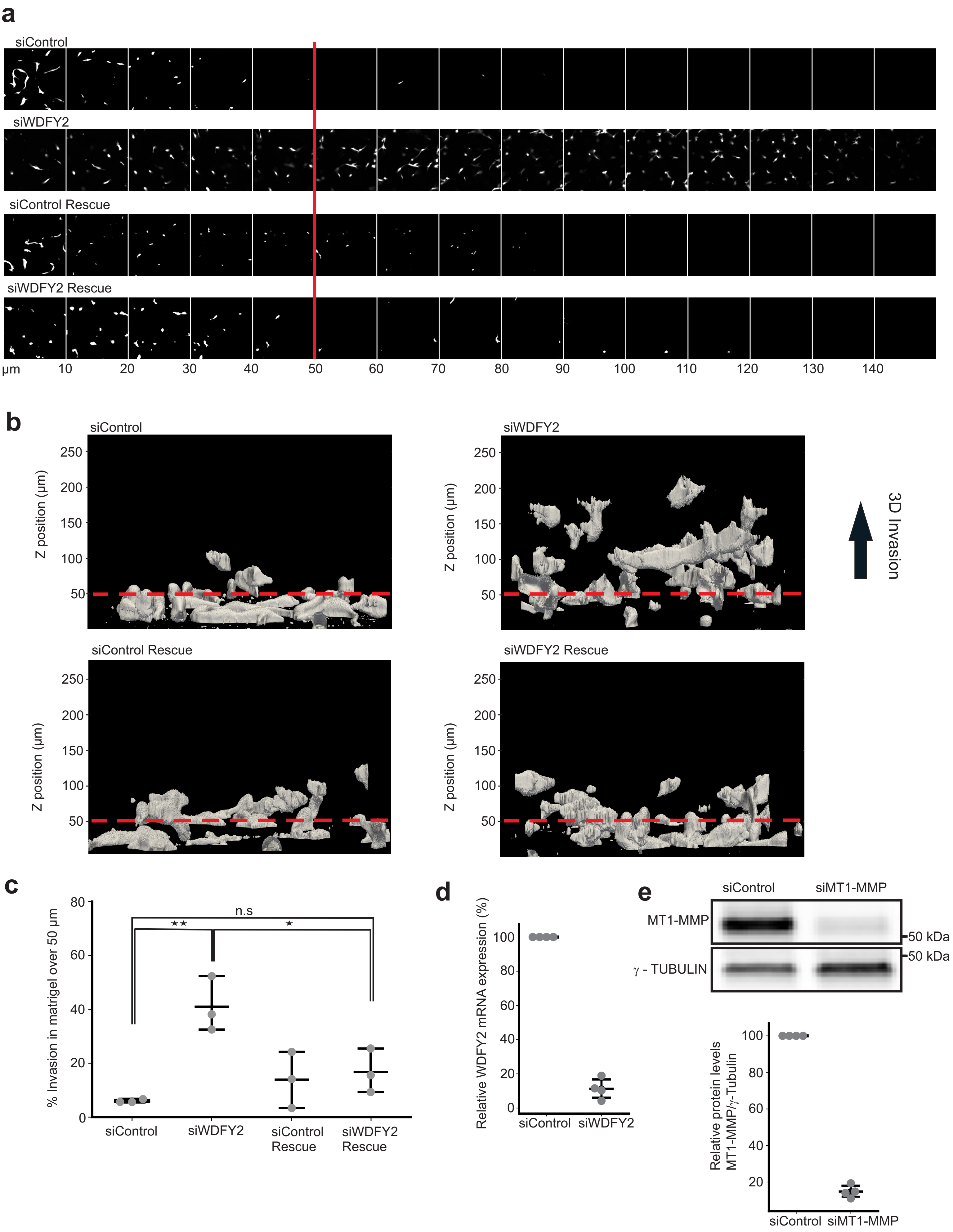

a
siControl
siWDFY2
siControl Rescue
siWDFY2 Rescue
µm
10
20
30
40
50
60
70
80
90
100
110
120
130
140
b
siControl
siWDFY2
siControl Rescue
siWDFY2 Rescue
Z position (µm)
250
200
150
100
50
3D Invasion
c
% Invasion in matrigel over 50 µm
80
60
40
20
0
n.s
siControl
siWDFY2
siControl Rescue
siWDFY2 Rescue
d
Relative WDFY2 mRNA expression (%)
100
80
60
40
20
0
siControl
siWDFY2
e
siControl
siMT1-MMP
MT1-MMP
γ - TUBULIN
50 kDa
50 kDa
Relative protein levels MT1-MMP/γ-Tubulin
100
80
60
40
20
siControl
siMT1-MMP

Supplementary Figure 7: Depletion of WDFY2 allows invasive cell migration in Matrigel™

a) Optical sections (10 µm) of RPE1 cells transfected with non-targeting siRNA (SiControl) and siRNA targeting WDFY2 (SiWDFY2) and RPE1 cells expressing siRNA resistant GFP-WDFY2 transfected with siRNA targeting WDFY2 (siWDFY2 rescue) invading fibronectin-supplemented Matrigel™. Cells were stained with Calcein-AM. The red line indicates the z-axis threshold (50 µm) defining invading cells.
b) Orthogonal view of a 3D reconstruction of RPE1 and RPE1 (WDFY2 rescue) cells treated as above. The red line indicates the z-axis threshold (50 µm) defining invading cells.
c) Quantification of invasion of RPE1 cells treated as above. Plotted data points indicate the mean of each experiement ,15 z-stacks per experiment, n= 3 experiments, also shown are the mean ± 95% CI. ANOVA with Bonferroni post-test, $p = 0.0047$. *$p < 0.05$, **$p < 0.01$, ***$p < 0.001$, n.s. not statistically significant.
d) Quantification of WDFY2 depletion by Realtime PCR. n= 4 experiments, also shown are the mean ± 95% CI.
e) Western blot showing the protein level of MT1-MMP after siRNA treatment. Graph showing quantification of western blots of relative MT1-MMP protein levels in relation to γ-Tubulin loading control and normalized to siControl ,n = 4 experiments. Shown are individual experiments and the mean ± 95%CI.

Source data are provided as a Source Data file.

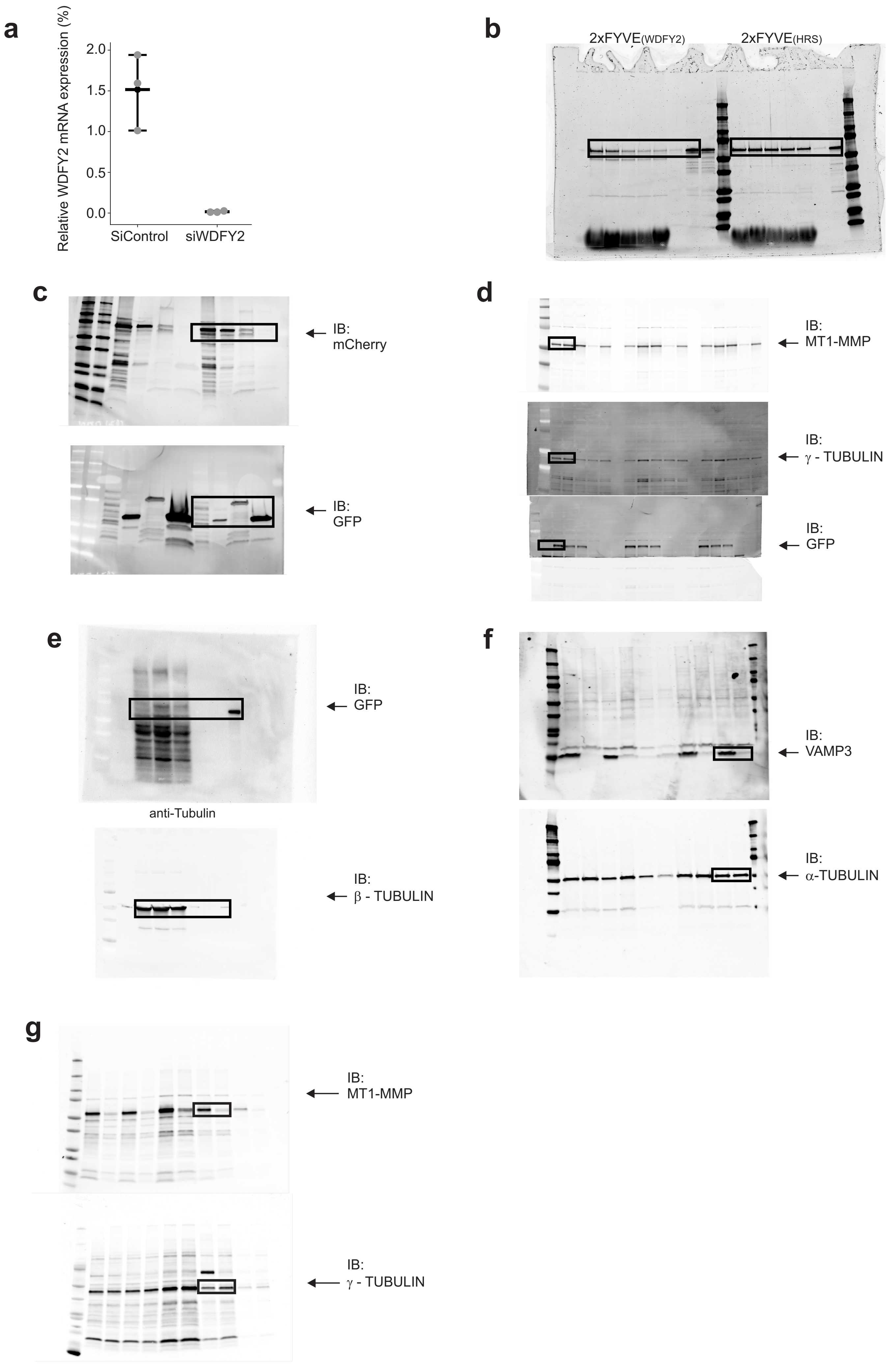

a
Relative WDFY2 mRNA expression (%)
2.0
1.5
1.0
0.5
0.0
SiControl
siWDFY2
b
2xFYVE(WDFY2)
2xFYVE(HRS)
c
IB: mCherry
IB: GFP
d
IB: MT1-MMP
IB: γ - TUBULIN
IB: GFP
e
IB: GFP
anti-Tubulin
IB: β - TUBULIN
f
IB: VAMP3
IB: α-TUBULIN
g
IB: MT1-MMP
IB: γ - TUBULIN

Supplementary Figure 8: Uncropped western blots

a) Quantification of WDFY2 depletion by Realtime PCR in MDA_MB231 cells. Shown are individual experiments and the mean ± 95%CI.
b) Uncropped gel stained with Coomassie in figure 3e. Boxed area indicates the cropped region.
c) Uncropped western blot in figure 4b. Boxed area indicates cropped region.
d) Uncropped western blots in figure 6g. Boxed area indicates cropped region.
e) Uncropped western blots in Supplementary Figure 1. Boxed area indicates cropped region.
f) Uncropped western blots in Supplementary Figure 6g. Boxed area indicates cropped region.
g) Uncropped western blots in Supplementary Figure 7. Boxed area indicates cropped region.

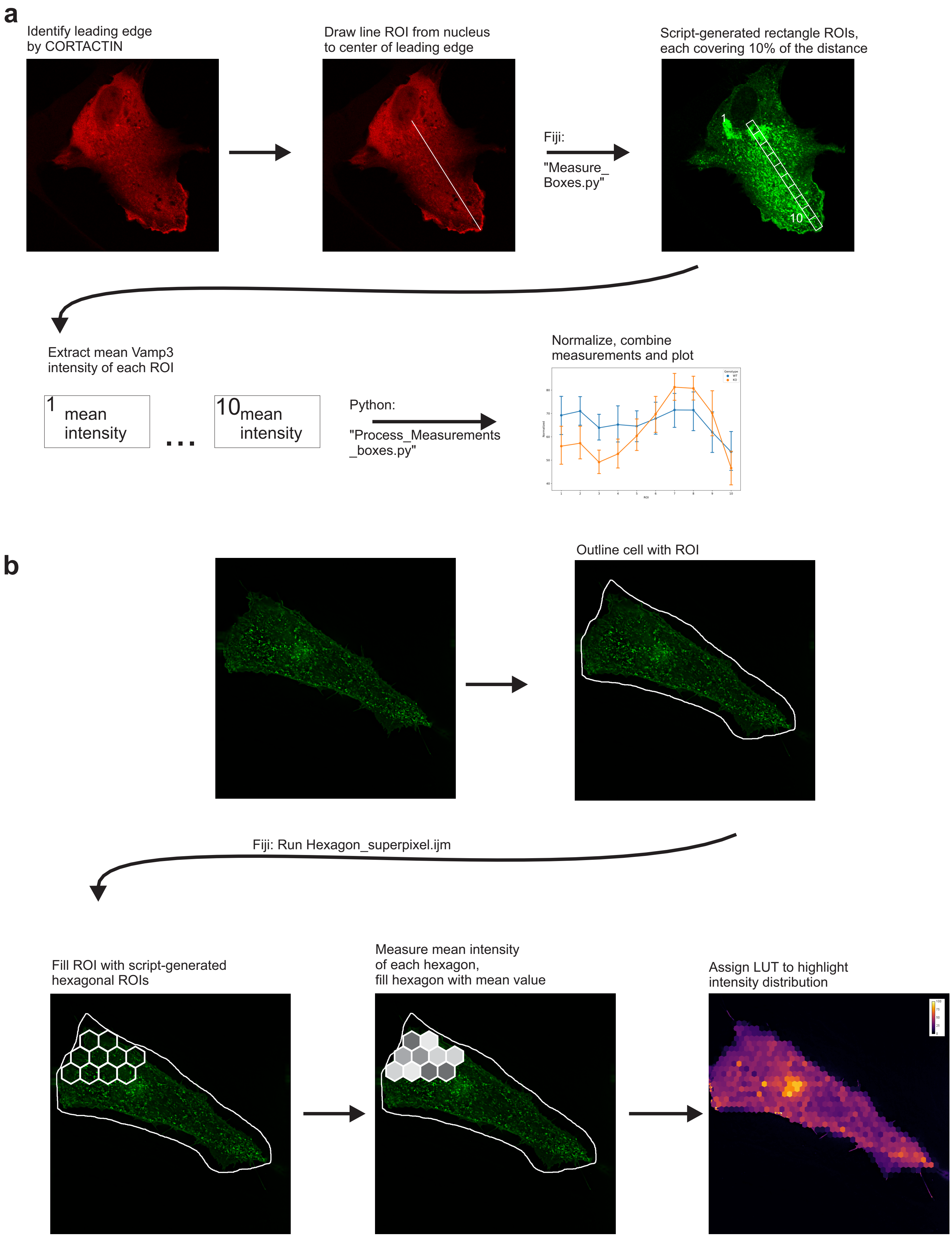
a
Identify leading edge
by CORTACTIN
Draw line ROI from nucleus
to center of leading edge
Fiji:
"Measure_
Boxes.py"
Script-generated rectangle ROIs,
each covering 10% of the distance
1
10
Extract mean Vamp3
intensity of each ROI
1 mean
intensity
...
10 mean
intensity
Python:
"Process_Measurements
_boxes.py"
Normalize, combine
measurements and plot
b
Outline cell with ROI
Fiji: Run Hexagon_superpixel.ijm
Fill ROI with script-generated
hexagonal ROIs
Measure mean intensity
of each hexagon,
fill hexagon with mean value
Assign LUT to highlight
intensity distribution

Supplementary Figure 9: Schematic overview of image processing/measurement steps

a) Schematic display of the processing steps to extract the data displayed in Figure 4b. Cells expressing GFP-Vamp3 were stained with CORTACTIN to identify leading edges. A line ROI was drawn between the rim of the nucleus and the leading edge, and rectangular ROIs, each covering 10% of the distance between the leading edge, were automatically generated using the Imagej script "Measure_Boxes.py" and their mean intensity in the Vamp3 channel was extracted. Mean intensities of all cells were normalized and plotted using the script "Process_Measurements_boxes.py" in Python.
b) Generation of superpixels to visualize Vamp3 distribution. Cells were outlined using ImageJ and an ImageJ macro (https://gist.github.com/mutterer/035ade419bf9c96475ce) was used to generate hexagonal ROIs. Mean intensity values for each hexagon were measured and the corresponding hexagon filled with the mean value, thereby generating superpixels. A false-color lookup table was applied to visualize intensities.

a

Figure	Experiment	Statistical test	What was tested?	p-Value	T-Value	DOF	F-value(ANOVA)
5d	Vamp3 secretion	T-Test (two-sided)	Means of 3 experiments	0.0021	-7.1246	4	n.a.
5e	Vamp3 in EEA1	T-Test (two-sided)	Means of 4 experiments, normalized (division by mean per experiment)	0.00015	8.3904	6	n.a.
5f	Vamp3 in Lamp1	T-Test (two-sided)	Means of 4 experiments, normalized (division by mean per experiment)	0.00083	6.1656	6	n.a.
6d	MT1-MMP secretion	T-Test (two-sided)	Means of 3 experiments	0.00265	-6.6482	4	n.a.
6f	WDFY2 OE and MT1-MMP recruitment	T-Test (two-sided)	Means of 3 experiments	0.02007	-3.7425	4	n.a.
6g	MT1-MMP secretion after Vamp3 KD	T-Test (two-sided)	Means of 3 experiments	0.01489	4.0962	4	n.a.
7c	Gelatin degradation RPE1	T-Test (two-sided)	Means of 4 experiments, normalized (division by mean per experiment)	0.00057	-6.6220	6	n.a.
7d	Gelatin degradation + MT1-MMP KD RPE1	T-Test (two-sided)	Means of 3 experiments, normalized (division by mean per experiment)	1.028e-07	87.3835	4	n.a.
7e	Gelatin degradation after Vamp3 KD	T-Test (two-sided)	Means of 3 experiments, normalized (division by mean per experiment)	0.00021	12.77329	4	n.a.
8c	Matrigel invasion KO	T-Test (two-sided)	Means of 3 experiments	0.01629	-3.9874	4	n.a.
8d	Knockout Collagen invasion	T-Test (two-sided)	Means of 3 experiments	0.03894	-3.0257	4	n.a.
9c	Invasion_MDA	Anova with Bonferroni post-test	Means of 3 experiments	ANOVA table	n.a.	11	11,31
9f	PC3-Invasion	T-Test (two-sided)	Means of 3 experiments	0.002965	6.4551	4	n.a.
S2c	Tubule length LatB	T-Test (two-sided)	Means of 3 experiments	0.000578	-9.9265	4	n.a.
S2d	Tubule length CK666	T-Test (two-sided)	Means of 3 experiments	0.004530	-5.7517	4	n.a.
S5f	Tubule length wt vs KO	T-Test (two-sided)	Means of 3 experiments	0.119316	1.9762	4	n.a.
S5g	Tubule lifetime wt vs ko	T-Test (two-sided)	Means of 3 experiments	0.000360	11.21440	4	n.a.
S6f	WDFY2 OE and MT1-MMP recruitment_WB	T-Test (two-sided)	Means of 3 experiments	0.11888	-1.8183	6	n.a.
S7c	Invasion_RPE1_RNAi	Anova with Bonferroni post-test	Means of 3 experiments	ANOVA table	n.a.	11	9,781
S5d	TfR recycling assays	Anova with Bonferroni post-test	Means of 3 experiments	ANOVA table	n.a.	17	151.9

b

Figure 8c: MDA-MB231 invasion					
Table Analyzed	MDA_RNAI_invasion				
One-way analysis of variance					
P value	0,0030				
P value summary	**				
Are means signif. different? (P < 0.05)	Yes				
Number of groups	4				
F	11,31				
R squared	0,8092				
ANOVA Table	SS	df	MS		
Treatment (between columns)	1744	3	581,4		
Residual (within columns)	411,2	8	51,40		
Total	2155	11			
Bonferroni's Multiple Comparison Test	Mean Diff.	t	Significant? P < 0.05?	Summary	95% CI of diff
MDA SCR vs MDA KD	-29,94	5,114	Yes	**	-50.30 to -9.570
MDA SCR vs Cherry SCR	-2,411	0,4119	No	ns	-22.78 to 17.95
MDA SCR vs Cherry KD	-4,645	0,7935	No	ns	-25.01 to 15.72
MDA KD vs Cherry SCR	27,52	4,702	Yes	**	7.159 to 47.89
MDA KD vs Cherry KD	25,29	4,320	Yes	*	4.925 to 45.65
Cherry SCR vs Cherry KD	-2,234	0,3816	No	ns	-22.60 to 18.13

c

Supplemental Figure 7c RPE1 invasion RNAi					
Table Analyzed	RPE1_RNAI_invasion				
One-way analysis of variance					
P value	0,0047				
P value summary	**				
Are means signif. different? (P < 0.05)	Yes				
Number of groups	4				
F	9,781				
R squared	0,7858				
ANOVA Table	SS	df	MS		
Treatment (between columns)	2047	3	682,5		
Residual (within columns)	558,2	8	69,78		
Total	2606	11			
Bonferroni's Multiple Comparison Test	Mean Diff.	t	Significant? P < 0.05?	Summary	95% CI of diff
SCR vs KD	-34,99	5,130	Yes	**	-58.72 to -11.26
SCR vs Overexp	-7,918	1,161	No	ns	-31.64 to 15.81
SCR vs Rescue	-10,80	1,584	No	ns	-34.53 to 12.93
KD vs Overexp	27,07	3,969	Yes	*	3.344 to 50.80
KD vs Rescue	24,19	3,546	Yes	*	0.4601 to 47.91
Overexp vs Rescue	-2,884	0,4229	No	ns	-26.61 to 20.84

d

Supplemental Figure 5d TfR recycling assay					
Table Analyzed	TfR recycling				
One-way analysis of variance					
P value	< 0.0001				
P value summary	***				
Are means signif. different? (P < 0.05)	Yes				
Number of groups	6				
F	151,9				
R squared	0,9844				
ANOVA Table	SS	df	MS		
Treatment (between columns)	1,433E+12	5	2,866E+11		
Residual (within columns)	22640000000	12	1887000000		
Total	1,455E+12	17			
Bonferroni's Multiple Comparison Test	Mean Diff.	t	Significant? P < 0.05?	Summary	95% CI of diff
WT vs KO	-9617	0,2712	No	ns	-108200 to 88960
Wt_10min vs KO_10min	-18400	0,5188	No	ns	-117000 to 80170
WT_30min vs KO_30min	-13020	0,3670	No	ns	-111600 to 85560

Supplementary Figure 10: Summary of statistical tests

a) Overview of statistical tests for each figure using these tests, including n, p-values, t-values, degrees of freedom and F-values for ANOVA
b) ANOVA table describing the statistics for Figure 8c
c) ANOVA table describing the statistics for Supplementary Figure 7c
d) ANOVA table describing the statistics for Supplementary Figure 5d
